# The Frequency of Specific *KRAS* Mutations, and Their Impact on Treatment Choice and Survival, in Patients With Metastatic Colorectal Cancer

**DOI:** 10.1093/oncolo/oyad117

**Published:** 2023-05-04

**Authors:** Ana Fernández Montes, Vicente Alonso Orduña, Elena Asensio Martínez, Nuria Rodríguez Salas, Esperanza Torres, Diego Cacho Lavín, Rosa María Rodríguez Alonso, Esther Falcó, Joan Carles Oliva, Lluis Cirera, Jesus García Gómez, Carles Pericay

**Affiliations:** Servicio de Oncología Médica, Complexo Hospitalario Universitario de Ourense, Calle Ramón Puga Noguerol, Ourense, Spain; Servicio de Oncología Médica, Hospital Universitario Miguel Servet, Instituto de Investigacion Sanitaria de Aragon, Paseo Isabel la Católica, Zaragoza, Spain; Servicio de Oncología Médica, Hospital General Universitario de Elche, Carrer Almazara, Elche, Alicante, Spain; Servicio de Oncología Médica, Hospital Universitario La Paz, Paseo de la Castellana, Madrid, Spain; UGC intercentros de Oncología Médica, Hospitales Universitarios Regional y Virgen de la Victoria and Instituto de Investigación Biomédica de Málaga (IBIMA), Campus de Teatinos, Málaga, Spain; Servicio de Oncología Médica, Hospital Universitario Marqués de Valdecilla, Santander, Cantabria, Spain; Servicio de Oncología Médica, Hospital Universitario Reina Sofía-Córdoba, Avenida Menéndez Pidal, Córdoba, Spain; Servicio de Oncología Médica, Hospital de Son Llàtzer, Carretera de Manacor, Palma de-Mallorca, Illes Balears, Spain; Institut d’Investigació I Innovació I3PT, Fundació Parc Taulí, Plaça Taulí, Sabadell, Barcelona, Spain; Servicio de Oncología Médica, Hospital Universitario Mútua Terrassa, Plaça del Doctor Robert, Terrassa, Barcelona, Spain; Servicio de Oncología Médica, Complexo Hospitalario Universitario de Ourense, Calle Ramón Puga Noguerol, Ourense, Spain; Servicio de Oncología Médica, Hospital Universitari Parc Taulí, Plaça Taulí, Sabadell, Barcelona, Spain

**Keywords:** colonic neoplasms, mutation, codon, genes (ras), prognosis

## Abstract

**Background:**

Patients with metastatic colorectal cancer (mCRC) and *KRAS* mutations have a poor prognosis, seemingly dependent on the location of the mutation. This multicenter, retrospective, cohort study assessed the frequency and prognostic value of specific *KRAS* mutation codon locations in mCRC patients, and survival outcomes in relation to treatment.

**Materials and Methods:**

Data from mCRC patients treated in 10 Spanish hospitals between January 2011 and December 2015 were analyzed. The main objective was to investigate (1) the impact of *KRAS* mutation location on overall survival (OS), and (2) the effect of targeted treatment plus metastasectomy and primary tumor location on OS in patients with *KRAS* mutations.

**Results:**

The *KRAS* mutation location was known for 337/2002 patients. Of these, 177 patients received chemotherapy only, 155 received bevacizumab plus chemotherapy, and 5 received anti-epidermal growth factor receptor therapy plus chemotherapy; 94 patients underwent surgery. The most frequent *KRAS* mutation locations were G12A (33.8%), G12D (21.4%), and G12V (21.4%). Compared with other locations, patients with a G12S mutation had the shortest median OS (10.3 [95% CI, 2.5-18.0] months). OS was longer in patients who underwent surgery versus those who did not, with a trend toward prolonged survival with bevacizumab (median OS 26.7 [95% CI, 21.8-31.7] months) versus chemotherapy alone (median OS 23.2 [95% CI, 19.4-27.0] months).

**Conclusion:**

These findings confirm that *KRAS* mutation location may predict survival outcomes in patients with mCRC, and suggest that pre-/post-operative bevacizumab plus metastasectomy provides survival benefits in patients with *KRAS* mutations.

## Teaser

Patients with metastatic colorectal cancer (mCRC) and KRAS mutations have a poor prognosis, seemingly dependent on the location of the mutation. This study assessed the frequency and prognostic value of specific KRAS mutation codon locations in mCRC and survival outcomes in relation to treatment.

**Implications for Practice**

Specific gene mutations, including *KRAS*, are linked to poor prognosis in metastatic colorectal cancer (mCRC) patients. We found that survival differs depending on the location of the *KRAS* mutation, with codon G12S mutations being associated with shorter median overall survival. We also confirmed that resection of metastases leads to increased survival, especially in patients treated with bevacizumab, a biologic that targets vascular endothelial growth factor A. Thus, G12S *KRAS* mutations appear to predict a poorer prognosis than other *KRAS* mutations. Metastases resection should be considered wherever possible in mCRC patients with *KRAS* mutations; the addition of pre-/post-operative bevacizumab to chemotherapy may improve overall survival.

## Introduction

Colorectal cancer (CRC) is the 3rd most common type of cancer and the second deadliest worldwide, causing the death of over 800 000 patients per year.^1,2^ According to a global report from 2018, almost 2 million people in the world have CRC.^1^ CRC is also the 4th ranked cause of cancer burden worldwide, after lung, liver, and stomach cancer.^2^ The highest incidence rates of CRC are found in Europe, Australia, and New Zealand, whilst the lowest have been reported in Africa and South Central Asia.^1^ If diagnosed and treated early, CRC can be cured; however, about 25% of CRC cases become metastatic.^3,4^ According to data from the SEER 18 registries database, released in November 2018, the relative 5-year survival rate is 14.5% in patients with metastatic CRC (mCRC), compared with 71.1% and 89.8% in those with regional and localized CRC, respectively.^5^ A prognostic model for prediction of survival has estimated the median overall survival (OS) in patients with mCRC at ~24-36 months, with the most relevant prognostic factors being age, number of organs affected by metastasis, primary tumor location (left or right colon), RAS-BRAF mutational status, and treatment modality.^6^

First-line treatments for mCRC include chemotherapy combinations usually with irinotecan or oxaliplatin; biologics targeting the vascular endothelial growth factor A (VEGF-A; ie, bevacizumab) or the epidermal growth factor receptor (EGFR) in combination with chemotherapy are also indicated as first-line treatments in patients without reduced organ function, poor performance status, or cardiovascular insufficiency.^7^ Of these, bevacizumab, but not anti-EGFR agents, is indicated for patients with mCRC who harbor a *KRAS* mutation.^7-9^ European guidelines recommend to first determine whether patients are eligible for tumor resection and to define the status of the resection of the primary tumor. In medically fit patients with metastatic disease, European guidelines also recommend tumor resection, when possible, even prior to pharmacologic treatment if patients have a resectable tumor and favorable prognostic criteria.^7^ Patients with unresectable tumors are eligible for conversion therapy, which aims to shrink the tumor so that it can be resected, as this has been shown to improve long-term effects and OS.^10^ However, there is currently no consensus regarding the optimal conversion treatment strategy, and chemotherapy with anti-EGFR or with bevacizumab is generally used based on the tumor mutational profile and the drug toxicity profile.^7,10^

Mutations in specific genes, including *KRAS*, have been linked to poor prognosis in patients with mCRC.^8,11-16^ Mutations in the gene for *KRAS* are some of the most prevalent gene mutations in patients with mCRC and are present in approximately 40% of patients with mCRC.^11,17^ The effect of *KRAS* mutations on prognosis appears to vary depending on the codon location of the mutation.^14,16,18^ For example, Imamura et al. found that *KRAS* mutations in codon 12 were associated with poorer outcomes compared with wild-type *KRAS*. In their study, the G12V mutation was associated with the highest mortality rates (multivariate hazard ratio [HR] 2.00; 95% CI, 1.38-2.90, *P* = .0003).^14^ Fiala et al. found that, besides patients with G12V mutations, those with G12A *KRAS* mutations died earlier (median overall survival [OS] 6.6 months [4.8-8.4]) than patients with tumors harboring other *KRAS* mutation types (median OS 11.6 months [9.0-14.3]; *P* < .001).^13^ In addition, results from Phipps et al. showed lower survival rates in patients with mutations in codon 13 compared with wild-type *KRAS*.^16^

The aim of this real-world study was to investigate the impact of *KRAS* mutations in specific codons on survival in patients receiving treatment for mCRC in Spain, the effect of combining metastatic surgery with bevacizumab treatment on OS in patients with *KRAS* mutations, and whether prognosis was affected by the primary tumor location in patients with *KRAS* mutations.

## Material and Methods

### Patients and Study Design

This multicenter, retrospective, cohort study included data from patients with mCRC treated in 10 Spanish hospitals between January 2011 and December 2015. Patients were included in the analysis if they had a histologically confirmed diagnosis of mCRC between January 2011 and December 2015 and were ≥18 years old at the time of the mCRC diagnosis; patients with primary metachronous malignant tumors diagnosed between January 2011 and December 2015 were also included; patients had to have received at least 1 cycle of chemotherapy as first-line treatment. Only patients whose clinical and medical records were available from the primary diagnosis and for at least 1 year following diagnosis were eligible. The following data were extracted from the patients’ clinical records: age, gender, *KRAS* mutation status and location, treatment (including surgery and targeted treatments), timing of targeted treatment in relation to surgery, and outcomes. Patients were excluded from the study if they had been diagnosed with secondary synchronous or metachronous malignant disease between January 2011 and December 2015, if they had been treated for mCRC before January 2011 unless they had undergone prior surgical resection of the primary tumor and/or received post-operative treatment following primary metastatic tumor resection, in which case they were considered eligible. Patients were excluded from this study if they had undergone metastatic tumor resection before January 2011.

The study was approved by the ethics committee of each hospital involved. The use of the data was approved by the Committee on the Ethics of Medicinal Products Research (study code 2017/551).

### Primary Outcomes

The main outcomes included progression-free survival (PFS), defined as the time from mCRC diagnosis to first detected progression, death or loss to follow-up, and overall survival (OS), defined as the time from mCRC diagnosis to death or loss to follow-up. PFS and OS were analyzed according to the exact codon location of the *KRAS* mutation, surgical treatment or not, by targeted first-line treatment versus non-targeted treatment, by preoperative versus postoperative versus perioperative treatment (ie, before versus after versus before and after surgery, respectively), and by primary tumor location.

### Statistical Analysis

Numerical data were reported as median (interquartile range [IQR]) or mean (95% CI). Data were compared using the chi-squared test.

OS was estimated using Kaplan-Meier analysis, by tumor location, metastatic tumor resection, treatment (targeted therapy versus non-targeted therapy), and primary tumor location, and compared between groups using the log-rank test and Cox regression analysis. All *P*-values < .05 (2-sided) were regarded as significant. Multivariate analysis was performed by adjusting for potential confounders (age, sex, chemotherapy, and presence/absence of surgery) in a Cox regression model. SPSS software package (IBM SPSS Statistics Version 25.0/2021) was used for statistical analysis.

## Results

### Study Population

Of the 2024 eligible patients with mCRC, 2002 met all inclusion criteria; of these, the exact codon location of the *KRAS* mutation was known for 337 patients. These patients had a median (IQR) age of 65.0 (32.2-87.4) years and 212 (62.9%) were males (Table 1).

**Table 1.** Clinical and demographic characteristics of patients, according to *KRAS* mutation codon location.

	*KRAS* mutation location						
	G12A	G12C	G12D	G12R	G12S	G12V	G13D
*N*, %	114 (33.8)	20 (5.9)	72 (21.4)	6 (1.8)	16 (4.7)	72 (21.4)	37 (11.0)
Median (range) age, years	65.2 (34.8-81.8)	59.5 (36.9-81.7)	64.7 (39.6-80.4)	61.4 (45.4-79.6)	58.2 (42.9-83.9)	67.4 (32.2-82.9)	64.4 (41.2-87.4)
Gender, *n* (%)							
Male	79 (69.3)	11 (55.0)	39 (54.2)	3 (50)	14 (87.5)	39 (54.2)	27 (73.0)
Female	35 (30.7)	9 (45.0)	33 (45.8)	3 (50)	2 (12.5)	33 (45.8)	10 (27.0)
Tumor location, *n* (%)^a^							
Left colon	87 (76.3)	12 (60.0)	42 (58.3)	5 (83.3)	10 (62.5)	48 (66.7)	26 (70.3)
Right colon	26 (22.8)	7 (35)	28 (38.9)	0 (0.0)	4 (25)	23 (31.9)	10 (27.0)
Surgery, *n* (%)	27 (23.7)	6 (30)	25 (34.7)	3 (50)	5 (31.2)	18 (25.0)	10 (27.0)
No surgery, *n* (%)	87 (76.3)	14 (70)	47 (65.3)	3 (50)	11 (68.8)	54 (75.0)	27 (73.0)
Site of metastasis, *n* (%)							
Liver	44 (38.6)	7 (35.0)	24 (33.3)	4 (66.7)	7 (43.8)	27 (37.5)	14 (37.8)
Ganglia + liver	3 (2.6)	1 (5.0)	3 (4.2)	0 (0.0)	0 (0.0)	4 (5.6)	0 (0.0)
Peritoneal + liver	11 (9.6)	0 (0.0)	6 (8.3)	1 (16.7)	1 (6.3)	8 (11.1)	1 (2.7)
Lung + liver	21 (18.4)	3 (15.0)	9 (12.5)	0 (0.0)	1 (6.3)	9 (12.5)	5 (13.5)
Peritoneal	7 (6.1)	2 (10.0)	7 (9.7)	0 (0.0)	3 (18.8)	4 (5.6)	4 (10.8)
Lung	6 (5.3)	2 (10.0)	6 (8.3)	0 (0.0)	1 (6.3)	7 (9.7)	2 (5.4)
Other	22 (14.3)	5 (25)	17 (23.6)	1 (16.7)	3 (18.8)	13 (18.1)	11 (29.7)

^a^Total number of patients per tumor location including patients whose tumor location was not specifically in the left or the right colon.

Abbreviations: *KRAS*, Kirsten rat sarcoma viral oncogene homolog; *N*, number of patients.

While the sigmoid colon (28.1%) and rectum (24.2%) were the most frequent locations of mCRC, the liver was the most common metastatic site (37.7%; Table 1). The most frequent *KRAS* mutation location was G12A (*n* = 114, 33.8%), followed by G12D (*n* = 72, 21.4%) and G12V (*n* = 72, 21.4%; Table 2).

**Table 2.** Progression-free survival (PFS) and overall survival (OS) by codon location of the *KRAS* mutation.

	Point survival (95% CI), months							
	All patients with a *KRAS* mutation (*n* = 337)	G12A (*n* = 114)	G12C (*n* = 20)	G12D (*n* = 72)	G12R (*n* = 6)	G12S (*n* = 16)	G12V (*n* =72)	G13D (*n* = 37)
PFS								
Median	10.4 (9.3-11.6)	9.7 (8.1-11.3)	7.1 (2.5-11.8)	10.0 (8.0-11.9)	7.8 (0.0-20.2)	8.4 (0.0-20.2)	12.4 (11.2-13.6)	11.7 (6.9-16.5)
Mean	16.6 (14.3-18.8)	15.3 (11.9-18.7)	16.9 (7.7-26.2)	13.7 (10.7-16.7)	13.2 (5.5-20.9)	16.3 (5.3-27.3)	18.9 (14.5-23.2)	12.8 (9.7-15.8)
OS								
Median	25.4 (22.8-28.0)	26.8 (21.9-31.6)	31.1 (21.4-40.8)	23.4 (19.1-27.8)	14.5 (0.0-39.0)	10.3 (2.5-18.0)	28.5 (22.5-34.5)	19.1 (13.0-25.2)
Mean	31.8 (29.0-34.7)	30.8 (26.7-34.9)	39.1 (28.7-49.5)	30.5 (24.8-36.3)	27.9 (10.0-45.8)	23.7 (9.4-38.0)	34.9 (29.5-40.3)	23.6 (18.3-28.9)
Patients who underwent surgery								
Median	46.5 (39.0-54.0)	47.5 (37.7-57.4)	—	37.9 (19.9-56.0)	—	25.8 (15.7-35.9)	—	33.3 (15.1-51.5)
Mean	50.2 (44.2-56.1)	52.4 (43.1-61.8)	65.6 (63.7-67.6)	44.8 (32.6-57.1)	47.1 (47.1-47.1)	31.3 (19.2-43.5)	54.4 (43.7-65.1)	34.6 (22.7-46.5)
Patients who did not undergo surgery								
Median	20.0 (17.1-22.8)	20.9 (17.1-24.7)	25.8 (15.9-35.6)	20.6 (15.2-25.9)	6.2 (4.8-7.7)	8.00 (4.9-11.1)	22.6 (14.4-30.9)	17.0 (13.2-20.9)
Mean	24.2 (21.8-26.6)	23.7 (20.5-26.8)	27.0 (17.5-36.4)	23.0 (18.8-27.2)	8.7 (3.0-14.4)	16.2 (0.9-31.5)	28.7 (23.4-34.0)	19.4 (14.5-24.3)
*P* value^a^	**<.001**	**<.001**	**.003**	**.002**	**.025**	.062	**<.001**	**.013**

^a^Log rank test comparing median OS for surgery vs no surgery subgroups within each codon mutation group (significant values are shown in bold).

—Due to the low number of patients having an event in the group it was not possible to calculate a median value.

Abbreviation: *KRAS*, Kirsten rat sarcoma viral oncogene homolog.

Among those with tumor mutation data, the primary tumor location was the left colon in the majority of patients (*n* = 230, 68.2%), while 98 patients (29.1%) had a primary tumor in the right colon and 9 patients had tumors located in both the left and the right colon (Table 1).

### Treatment

**Figure 1.**
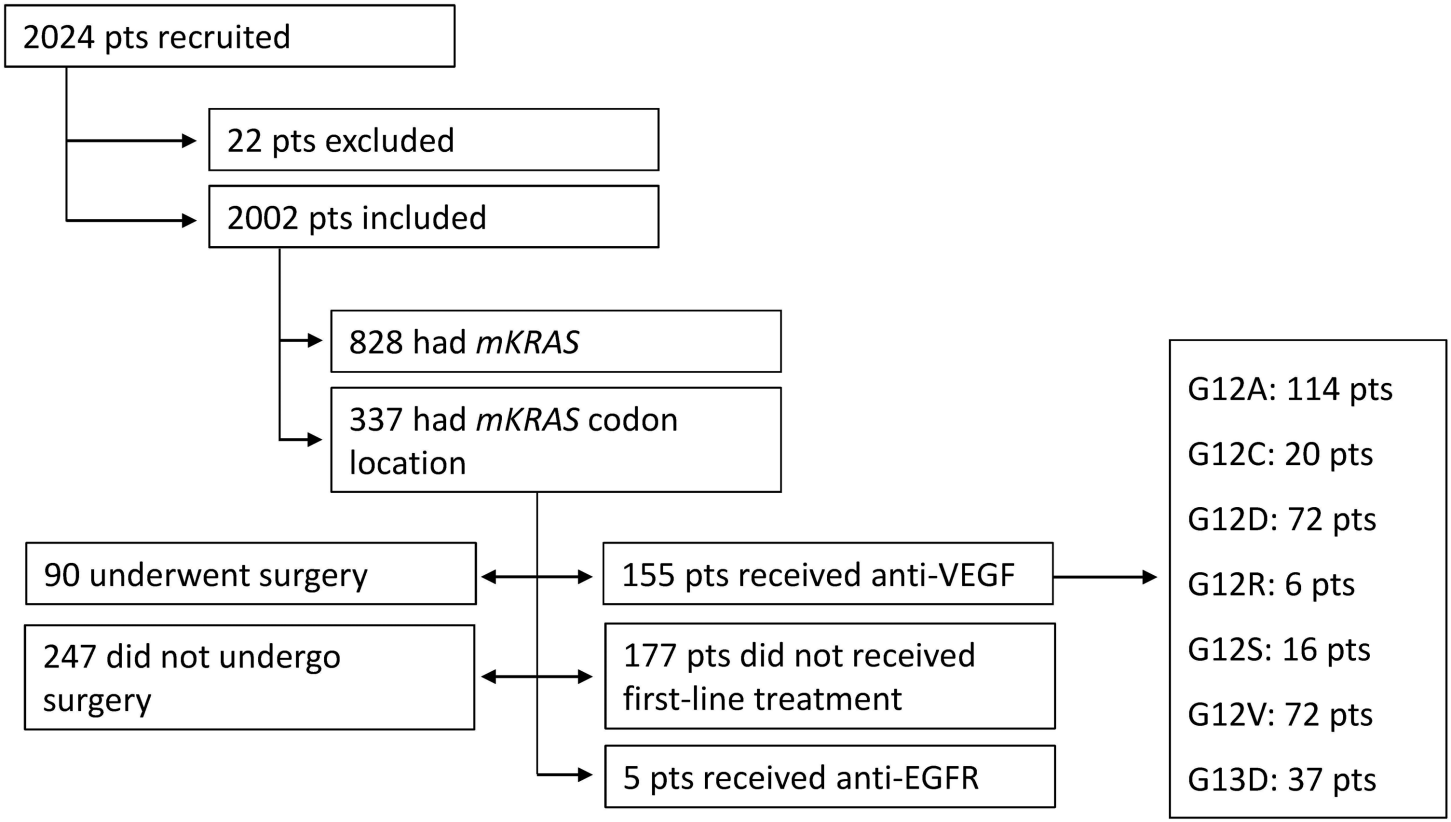
Study design and patient distribution. Abbreviations: EGFR, endothelial growth factor receptor; *mKRAS*, mutated V-Ki-ras2 Kirsten rat sarcoma viral oncogene homolog; pts, patients; VEGF, vascular endothelial growth factor.

**Figure 2.**
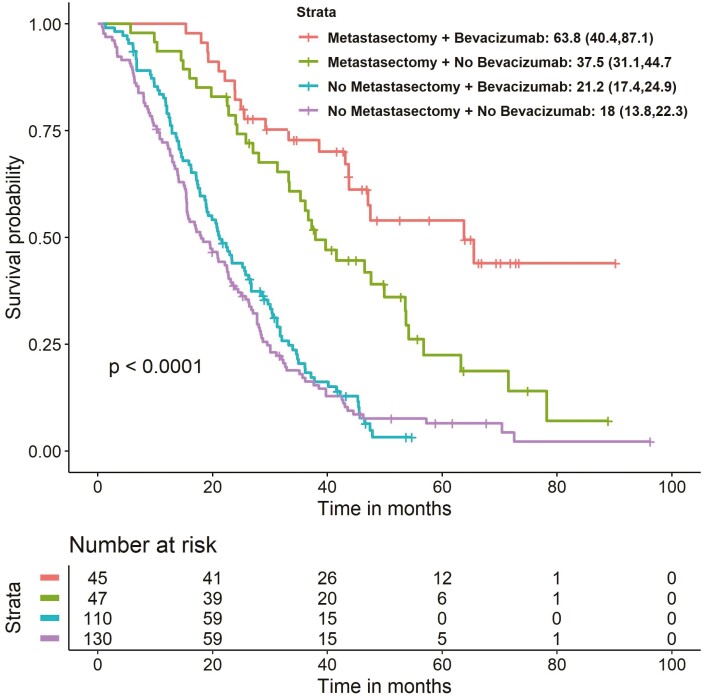
Kaplan-Meier analysis of overall survival by treatment. ^a^Data from patients treated with anti-EGFR (*n* = 5) were not analyzed by treatment or surgery. Abbreviations: EGFR, endothelial growth factor receptor; OS, overall survival; VEGF, vascular endothelial growth factor.

Of the 337 patients with a *KRAS* mutation, 177 did not receive targeted therapy, 155 received first-line targeted therapy with anti-VEGF (bevacizumab), and 5 received anti-EGFR therapy. All patients received chemotherapy. A total of 90 patients underwent surgery and 247 patients did not (Fig. 1). Of the 155 patients treated with anti-VEGF, 45 underwent surgical resection (29.0%) compared with 47 of the 177 patients (26.6%) who did not receive first-line targeted treatment (Fig. 2). Of the 45 patients who underwent surgery and received anti-VEGF therapy, 7 received only pre-operative anti-VEGF therapy and 38 received either post-operative anti-VEGF therapy only or both pre- and post-operative therapy.

### Primary Outcomes

Median OS in the 337 patients with *KRAS* mutations was 25.4 [95% CI, 22.8-28.0] months (Table 2). Of the 337 patients with a known *KRAS* mutation codon location, those with a G12S mutation (*n* = 16) had the poorest prognosis, with a median OS of 10.3 [95% CI, 2.5-18.0] months, while patients with a G12C *KRAS* mutation (*n* = 20) had the longest OS (31.1 [95% CI, 21.4-40.8] months). Median OS survival was longer in all patients who had undergone metastatic tumor resection than in those who did not undergo surgery, independently of the codon location of the *KRAS* mutation or the primary tumor location. The difference between surgery versus no surgery was statistically significant in subgroups with a *KRAS* mutation in codons G12A (*P* < .001), G12C (*P*= .003), G12D (*P* = .002), G12R (*P* = .025), G12V (*P* < .001), and G13D (*P* = .013) (Table 2). PFS was similar in all patients irrespective of the location of the *KRAS* mutation (*P* = .570), although patients with the mutation located in G12V had the longest numerical PFS (12.4 [95% CI, 11.2-13.6] months), while the shortest PFS was found in patients with a mutation at G12C (7.1 [95% CI, 2.5-11.8] months) (Table 2).

There was a trend toward longer survival in patients receiving first-line anti-VEGF therapy (*n* = 155; median OS 26.7 [95% CI, 21.8-31.7] months) than in patients treated with anti-EGFR (*n* = 5; median OS 25.4 [95% CI, 12.6-38.1] months) or not treated with targeted agents (*n* = 177; median OS 23.2 [95% CI, 19.4-27.0] months; *P* = .077). Irrespective of whether patients were treated with first-line anti-VEGF or non-targeted first-line treatment, all patients who underwent surgery had a significantly longer OS (*P* < .001 in both groups) (Fig. 2). The median OS was 63.8 [95% CI, 40.4-87.2] months in patients treated with anti-VEGF and surgery (*n* = 45), 21.2 [95% CI, 17.4-24.9] months in those treated with anti-VEGF but without undergoing metastatic surgery (*n* = 110), and 37.9 [95% CI, 31.1-44.7] months in patients who did not receive first-line targeted therapy but had a metastatic surgical resection (*n* = 47) (Fig. 2). Among patients treated with bevacizumab who did not undergo metastasectomy, those who had the primary tumor located in the left colon had longer median OS (23.4 [95% CI, 17.7-29.1] months) than patients whose primary tumor was located on the right (17.2 [95% CI, 13.6-20.7] months) (*P* = .001). Median PFS did not significantly differ based on the location of the primary tumor (Table 3).

**Table 3.** Median overall survival (OS) and progression-free survival (PFS) depending on the primary tumor location (left or right colon) and treatment (metastasectomy or not, chemotherapy ± bevacizumab).

	Median survival (95% CI), months		*P*-value
	Left colon	Right colon	
OS			
Metastasectomy, all patients	43.8 (34.5-53.1)	65.5 (36.7-94.3)	.133
Metastasectomy + bevacizumab	47.5 (26.1-69.0)	65.5 (25.2-105.8)	.415
Metastasectomy + no bevacizumab	37.9 (31.5-44.4)	56.8 (7.9-105.6)	.294
No metastasectomy + no bevacizumab	22.2 (18.9-25.4)	15.5 (14.6-16.5)	.731
Bevacizumab + no metastasectomy	23.4 (17.7-29.1)	17.2 (13.6-20.7)	**.011**
PFS			
Metastasectomy, all patients	16.3 (11.3-21.2)	16.5 (2.6-30.4)	.250
Metastasectomy + bevacizumab	18.0 (14.5-21.5)	29.7 (15.1-44.3)	.315
Metastasectomy + no bevacizumab	11.8 (9.7-14.0)	13.0 (12.6-13.4)	.593
No metastasectomy + no bevacizumab	8.3 (6.9-9.7)	5.5 (2.8-8.3)	.078
Bevacizumab + no metastasectomy	11.5 (9.6-13.5)	9.8 (7.9-11.7)	.138

There was a trend toward a longer median OS in patients receiving post-operative anti-VEGF therapy ± pre-operative anti-VEGF (65.5 [95% CI, 54.2-73.9] months; *n* = 38) compared with patients receiving only pre-operative anti-VEGF therapy (25.5 [95% CI, 20.8-55.6] months; *n* = 7) (*P* = .057).

## Discussion

This retrospective study in patients with mCRC showed that median OS differs depending on the codon location of the *KRAS* mutation (median OS 31.1 [95% CI, 21.4-40.8] months for G12C vs. 10.3 [95% CI, 2.5-18.0] months for G12S; Table 1) and that resection of metastases increases patient survival, especially in patients receiving bevacizumab (Fig. 2). In addition, post-operative bevacizumab treatment showed a trend toward prolonged OS compared with pre-operative bevacizumab treatment. These results show that the codon location of the *KRAS* mutation is an important prognostic factor in patients with mCRC. They also showed a trend toward prolonged OS when anti-VEGF therapy was combined with metastasectomy in patients with *KRAS* mutations.

Regarding the prognostic value of the codon location of the *KRAS* mutation, our results show that mutations at G12S were associated with the shortest OS duration, followed by those located at G12R and G13D. The finding that patients with *KRAS* mutations at G12S and G12R have a poor prognosis is consistent with previous studies by Zocche et al.^19^ and Imamura et al.,^14^ respectively, but is in contrast with other studies, which have found worse survival outcomes in patients with *KRAS* mutations at G12V and G12A.^13,14^ The difference between our results and those of previous studies may be partly due to the low number of patients in some of our patient groups, specifically those with G12R (*n* = 6) and G12S (*n* = 16) *KRAS* mutations. As such, our findings should be verified in a larger cohort of patients.

The most common codon locations of the *KRAS* mutation in our study were G12A (33.8%), G12D (21.4%), and G12V (21.4%) (Table 1). These results are mostly in line with previous studies on the location of the *KRAS* mutation, in which mutations at G12D and G12V were present at a higher frequency than mutations at other codon locations.^13,14,20,21^ In our study, the frequency of mutation at G12A was higher than in previous studies, which reported a G12A frequency of between 2.4% and 4.4%.^13,14,20,21^ These differences could be due to the geographic location of data collection and the year these data were analyzed since the incidence of CRC varies between countries and over time.^2^ Overall, when compared with patients with *KRAS* wild-type tumors, all patients with tumors harboring *KRAS* mutations have poorer survival outcomes, irrespective of the codon location.^13-16,20^

To date, no randomized controlled trials have evaluated the treatment efficacy of pre- or post-operative anti-VEGF plus chemotherapy; therefore, optimal treatment in mCRC patients undergoing resection of metastatic disease remains unknown.^22-24^ However, a systematic review and meta-analysis, as well as a phase II single-center study, have shown that pre-operative bevacizumab plus chemotherapy led to increased pathologic response that was associated with better survival outcomes in patients with mCRC.^22,24^ Together with the results from our study, these data highlight the need to evaluate treatment efficacy of pre-operative ± post-operative therapies in patients with mCRC.

Another important finding of this study was the impact of surgery on patient survival. Independent of the codon location of the *KRAS* mutation or whether or not patients were being treated with targeted therapies, all patients who underwent surgery (*n* = 94) had higher survival rates than patients who did not have surgery (*n* = 243). These results confirm what is already known regarding the positive impact of metastasis resection on patient survival.^25^ Of the patients who underwent surgery, patients with *KRAS* mutations at G12C, G12V, and G12A had the longest mean OS, and patients treated with bevacizumab had the longest median OS, whereas patients with a *KRAS* mutation at G12R, G12S or G13D and those who did not receive bevacizumab had the shortest median OS (Table 2; Fig. 2).

Overall, the results of our study suggest that metastatic tumor resection, especially when combined with post-operative (±pre-operative) bevacizumab (median OS, 63.8 [95% CI, 40.4-87.2] months) provides survival benefits. However, this treatment strategy does not seem to be a common practice, despite European guideline recommendations,^7^ because fewer than one-third of the patients in our study underwent surgery (Fig. 2). Our findings are consistent with a German study, in which patients who underwent tumor resection had a better 5-year survival rate than those who did not undergo surgery.^26^ Similarly, a Dutch study of survival trends over time found that an increase in survival rates was linked to an increased frequency of metastasectomy.^4^ Similar to our study, these previous studies reported that, although increasing over time, the overall number of tumor resections was low.

When considering primary tumor location, a significant difference in median OS between right and left colon tumors was only observed in patients who did not undergo surgery and were treated with bevacizumab, with a significantly shorter median OS in those with right- versus left-sided tumors (17.2 [95% CI, 13.6-20.7] months vs. 23.4 [95% CI, 17.7-29.1] months, respectively; *P* = .011). The lack of statistical significance in OS or PFS between right- and left-sided tumors among patients who underwent surgery (with or without bevacizumab treatment) in our study could have been due to an imbalance in the population size between left- and right-sided tumor groups (ie, the number of patients with left-sided tumors was more than 2.3 times the number with right-sided tumors). Right-sided tumors are known to be associated with a poorer prognosis than left-sided tumors in patients with mCRC.^27^ For instance, a post hoc analysis of the OPTIMOX3 DREAM phase III study on the prognostic value of primary tumor location in *KRAS* mutant mCRC showed that patients with right-sided tumors had a numerically shorter median OS than patients with left-sided tumors (19.4 [95% CI, 15.8-22.0] months vs. 24.9 [95% CI 22.5-30.0] months, respectively).^17^ Taken together with our results, these data suggest that a better understanding of the relationship between patient prognosis and tumor location might help to elucidate the mechanisms of colon carcinogenesis with implications for clinical and pharmacological research.

The results of our study should be interpreted with caution due to the retrospective nature of the analysis, especially because we evaluated only the impact of first-line treatment on survival, and did not consider the full treatment sequence that patients received. Another limitation is that widespread *KRAS* mutation testing was not well established at the start of the study period (ie, 2011) and data regarding the specific codon location were often not provided. These factors resulted in a relatively small number of patients with known *KRAS* codon location information. In addition, there was uneven sample distribution between treatments and the codon location of the *KRAS* mutation, resulting in only a small number of patients in some subgroups and a different proportion of patients with left- versus right-sided tumors. Finally, the retrospective nature of the study design and the small number of patients undergoing resection of metastasis were confounding variables with regard to the possible survival benefits of pre- and post-operative bevacizumab treatment; therefore, these benefits should be confirmed in a larger prospective study.

## Conclusion

Our retrospective study confirms that *KRAS* mutations at specific codon locations may predict survival outcomes in patients with mCRC. Our data also suggest that pre- and post-operative bevacizumab treatment, in addition to metastatic tumor resection, may provide survival benefits in patients with mCRC and *KRAS* mutations and should therefore be considered in this setting. Further improvement in survival rates might be possible by increasing the number of metastatic surgeries performed in patients with mCRC, as well as further studies of other treatment strategies that target *KRAS* mutations.

## Data Availability

The data underlying this article will be shared on reasonable request to the corresponding author.
